# Workload disparities and their role in the health of migrants and natives in Germany

**DOI:** 10.1186/s12889-024-19606-3

**Published:** 2024-08-09

**Authors:** Kai Ingwersen, Stephan L. Thomsen

**Affiliations:** Institute of Economic Policy, Leibniz University Hannover, Königsworther Platz 1, D-30167 Hannover, Germany

**Keywords:** Workload, Working conditions, Migrants, Self-reported health, BIBB/BAuA labour force survey

## Abstract

**Background:**

This study explores the health status differences between migrants and native Germans, focusing on potential disparities in their workloads. Physical and mental workloads can negatively impact individual health. Since various occupations come with distinct health-related patterns, occupational selection may contribute to systematic health disparities among socio-economic groups. Given the generally poorer health of migrants, they might experience systematic workload differences overall.

**Methods:**

We suggest a conceptual framework for the empirical analysis based on the theory of health as a durable good with health consumption and health investment as key parameters. We quantify the role of work tasks, job requirements and working conditions on individual health based on detailed information from the BIBB/BAuA labour force survey 2012 and 2018.

**Results:**

The empirical results reveal that migrants, i.e. foreigners and German citizens with a migration background, have a higher perception of workload and related health afflictions within the same occupation. Native Germans, on the other hand, experience a higher burden by high job requirements, both physically and mentally. The findings imply heterogeneous health impacts of work for migrants and native Germans due to differences in health consumption.

**Conclusions:**

The analysis shows that migrants report worse health than natives, with stronger negative effects of work-related conditions on their health, both physically and mentally. Women, in general, report poorer health conditions than men. The findings emphasize the importance of promoting human capital to reduce economic and health disparities, though caution is advised regarding affirmative actions for migrants; further research is needed to understand the underlying mechanisms and address these issues effectively.

**Supplementary Information:**

The online version contains supplementary material available at 10.1186/s12889-024-19606-3.

## Background

Over the last six decades, immigrants and their children have become part of the German society which is also reflected by a large and growing share of the labour force. However, differences remain and persons with a migration background, i.e., immigrants and their descendants, on average, still pursue occupations that demand lower qualifications, require higher physical strain, and are less well paid [1–4]. The selection of persons with a migration background into certain occupations as well as into tasks within these occupations lead to specific demands on this group compared to natives. This segmentation of the labour market may be the cause of the overall poorer health status – at least partially [2, 5, 6], as workload contributes directly to socio-economic differences in health status [7]. Good health contributes directly to reducing social inequalities and thus creating equal opportunities [6]. Therefore, work and working conditions are fundamental reasons for those (health) inequalities within and across generations [8]. For instance, workers in precarious jobs are particularly affected by stressful working conditions that are detrimental to their health, and their comparatively low pay for these jobs also limits the necessary investment in their own health (e.g. due to the spending for necessary expenses to keep a living) [9, 10]. In addition to a low social status, a poorer state of health comes on top and reinforces or even exaggerates social inequalities and segregation. However, besides labour market segmentation, there are also indications that even within the same occupation, persons with a migration background are exposed to a comparatively higher health burden [2, 11].

This study explores whether (and to what extent) migrants are exposed to higher workloads than native Germans even if they work in the same occupation and the same job position, and thus may have a lower health status. Given the overall poorer health of the migrant population, they may face systematic workload differences in the labour market in general. Since migrants are overproportionally found in precarious employment [12, 13], they possess a higher risk of health, economic and social decline. Thus, it is in the interest of society and government to systematically reduce health inequalities of the population for strengthening both economic performance and social life.

Individual health has played a focal role in numerous economic studies for many decades. As part of human capital, health is fundamental for being able to exert acquired qualifications optimally, exploit potential productivity and influence economic growth [14]. As a pioneer, Grossman [9] drafted an economic theory of individual health behaviour. He proposed a model in which health is a durable good that can be consumed and invested in. In addition to ageing, health consumption takes place through work, demanding leisure activities or an exhausting lifestyle. The health capital stock can be enhanced through investments in preventive healthcare, convalescence, recreation or the use of medical services [15, 16]. Consumption and investment in health are mutually dependent: While health is consumed by work, work is also required to generate income, which is necessary for health investment to maintain an adequate level of health. In that sense, physically demanding work requires a higher consumption of health. To maintain employability and productivity, health investments of the same magnitude are necessary. However, occupations with predominantly high physical demands often yield only a small income. This implies a stronger depreciation of health capital [17–19], as the low income is required for other basic needs (especially food and housing), postponing investments in health temporarily or even permanently. Since poor health and a small income reduce the quality of life and limit social participation, people of low socio-economic status, which, e.g., migrants often possess, are particularly threatened by social decline and work-related health difficulties. Therefore, reducing health gaps within the population will foster convergence in income distribution [20] and will reduce social inequality.

Empirical studies provide several health-influencing factors that directly result from work: Job tasks, special work requirements that include the working conditions, and the working climate have been shown to be relevant. According to Bellmann and Hübler [21], however, detailed job characteristics are all too often disregarded in the empirical health literature. Thus, occupational characteristics, decision-making competence, physical effort, environmental conditions, time pressure and multitasking all influence individuals’ health. In contrast, a higher wage allows individuals to take advantage of health services and preventive healthcare [4, 18, 22, 23]. Furthermore, the relationship between education and health is well depicted by Burgard and Lin [8]: Low-skilled workers are comparatively more often confronted with physically demanding jobs, which can cause both physical and psychological complaints. For well-educated people, physical demands are usually lower, however, higher educated workers are also more exposed to the risk of psychosocial stress, which – due to a high degree of permeability – also increases the risk of a negative spillover into private life [8]. In addition, the level of education attained not only influences the job and the tasks to be performed but also has an indirect effect on health and risk behaviours and how to deal with stress [21]. In the private environment, there are health-promoting but also factors that are detrimental to health. Studies by Cottini [23] and Giannoni et al. [18] show that living with a partner is positively related to health, whereas the presence of children worsens the condition due to increased load (e.g., care obligations, less leisure time for recovery) and broughtin diseases.

Although the health system in Germany ensures general medical care for everyone, access is not equal for all. In particular, healthcare utilisation for migrants can be more difficult due to cultural and language barriers [5]. For instance, being able to speak German well, has a positive effect on health via two channels: first, access to certain occupations and, second, easier access to medical care [24]. Further, asylum seekers and irregular migrants do not have the same access to health services as natives due to the different entitlements associated with their legal status, resulting in comparatively worse health of this group of migrants [25, 26].

Other reasons for divergent health investments of migrants are fundamental differences in behaviour, which are reflected in preventive healthcare or in the lower frequency of visiting a doctor. Both work-related and individual factors lead to the fact that people with lower levels of education, in physically demanding jobs and with lower job positions − i.e., usually people with a low social status − tend to be more exposed to a health-impairing environment. This includes in particular migrants, who are disproportionately often part of this social stratum. A relatively higher workload for an already disadvantaged group may promote health disparities and can exaggerate social inequality and segregation. Related empirical analyses of Oldenburg et al. [11] and Becker and Faller [2] support this perception reporting that employees with a migration background in Germany are more frequently exposed to physical stress. Wengler [24] confirms the lower health status of immigrants from Turkey in Germany. However, if socio-economic and individual characteristics are taken into account, the differences in health between immigrants and native Germans disappear. Hence, we can expect differences in perception of health and workload [27].

To examine differences in health status between migrants and native Germans for possible disparities in their workload, we provide a careful empirical description of occupations and related health complaints for migrants and native Germans in the German labour market. For this purpose, we use rich data from the 2012 and 2018 BIBB/BAuA Labour Force Survey. These data contain comprehensive information on individuals’ health, work tasks, job requirements, and working conditions for measuring workload and workload differences. We justify our choice of variables in the empirical model based on theoretical considerations following the literature. We model health as a durable good (initiated by Grossmann [9]; and extended by several researchers since then, e.g. by [10, 16]) which can be consumed and invested in. In this respect, besides ageing (which leads to a loss of initial health), socio-economic and work-related aspects (can) accelerate or delay this process. Socio-economic groups may differ in these aspects – maybe due to external reasons/constraints or behavioural differences.

Based on this reasoning, we suggest a conceptual framework for an integrated analysis of the confounding factors. In the following empirical analysis, we estimate the influence of a wide set of occupational and socio-economic factors on individual health at work providing insights on health consumption and investment patterns. Moreover, we consider a further distinction into the two subdomains physical health and mental health to reveal potential heterogeneity with respect to certain types of health afflictions. While comparing migrants and natives as the main distinction, we differentiate further by gender, as men and women have different perceptions of health and workload.

## Methods

### Approach

**Fig. 1:**
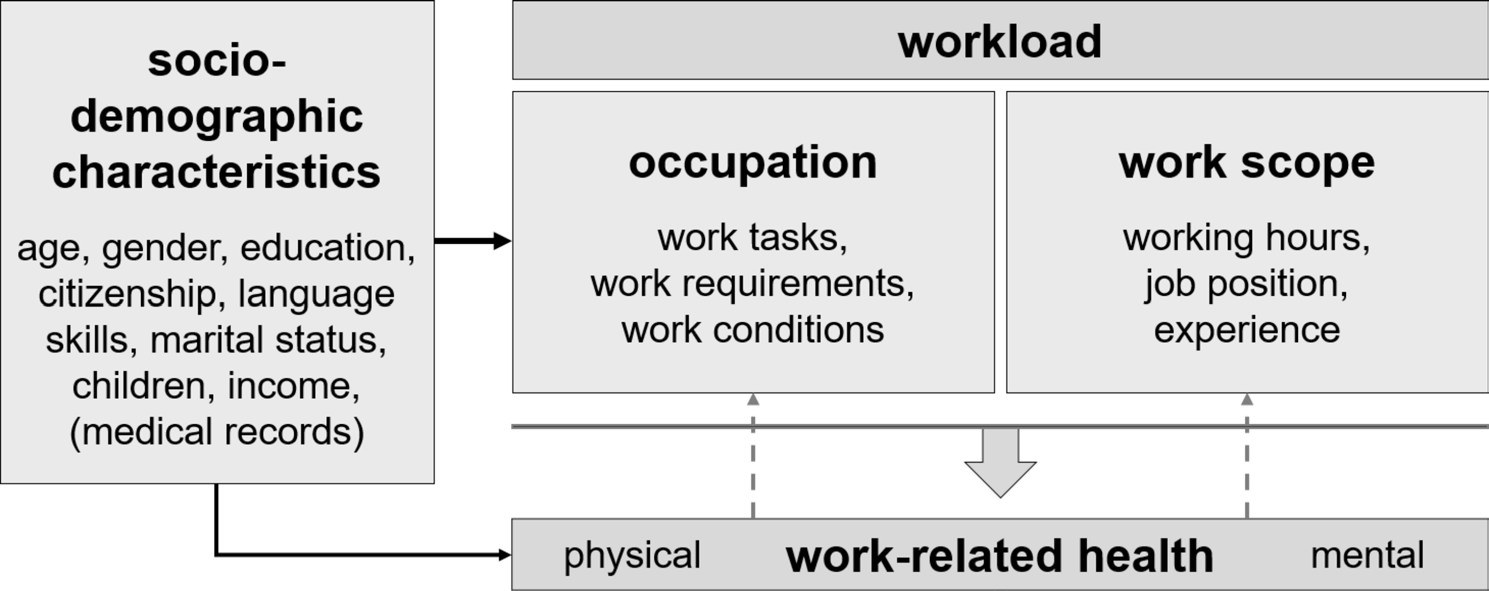
Schematic outline of the factors influencing work-related health. *Source*: Own illustration

While the available literature focuses on certain aspects, our aim is to bring together the different strands of health-influencing factors into an integrated analysis. For this purpose, we suggest a framework that depicts the relationship between workload and associated health. We examine the extent to which workplace-related stress affects individual health, taking relevant socio-demographic characteristics into account. The derivation of the framework is based on findings on workload shown in the literature. Figure 1 outlines the framework in its key references. Our empirical analysis initially focuses solely on the direction of the effect of workload on associated health. We are aware, of course, that the state of health of a person itself has an impact on the occupation and the tasks related to it but abstract from this in the current setting. In a dynamic context (for analysing developments), both directions of impact should be considered.

In this section, we will explain and justify the components of the conceptual framework and will describe how they are operationalized. The type of workload is determined by the occupation performed with its tasks, requirements, and working conditions, while the strength of workload is determined by the scope of work. Moreover, socio-demographic characteristics affect the health of a working person in two ways: Firstly, a person’s socio-demographic characteristics have a significant influence on his or her occupational choice (e.g., educational level). Secondly, these characteristics are fundamental determinants of occupational choice and the associated workload (see Fig. 1). As there are differences in health perception and reporting [28] and differences in workload assessment between men and women [29], we differentiate all our analyses by gender. Women rate tasks as more demanding than their male counterparts. This is also reflected in a gender-specific attitude toward specific tasks [29]. Based on this approach, differences in workload determinants can be identified according to gender and migration background, as well as in regards to their effects on health.

In order to transfer the suggested framework into an empirical analysis, operationalization of, first, the work-related determinants and, second, health is necessary. Generally, “*health is a state of complete physical*,* mental and social well-being and not merely the absence of disease or infirmity*” [30]. Since the individual’s state of health can usually only be described indirectly by the presence of ailments or complaints and accordingly provides only a limited picture of true health, we use self-reported health of workers. Self-reporting on health is a common and validated procedure in a large number of scientific papers, see besides others Cottini [17, 23], Dunn and Dyck [31] or Giannoni et al. [18]. Nonetheless, population groups may perceive and assess health differently [27], resulting in group-specific health patterns. Therefore, differences in perception of health and actual health can be assumed. For this reason, we will use both self-reported health status and an approach with the level of work-related complaints to allow for a comprehensive depiction of health.

Furthermore, occupations differ considerably in their health demands, so a detailed consideration is essential for our empirical analysis. Kroll et al. [32, p. 2] note that “*work-related stress results from environmental stress*,* physical stress*,* and psychological and social stress*”. We follow this distinction and subdivide the job characteristics into three central groups of factors (see Table 1 for an overview): (1) *work tasks* are the activities performed within the job, (2) *job requirements* depict specifications and work performance, and (3) *working conditions* describe the work environment and the working atmosphere. With regard to the “job demands-resources model” (JDR model) by Demerouti et al. [33] and a special focus on psychological health problems, it is appropriate to divide working conditions into factors that put a strain on work demands (e.g., job pressure) and on work resources that cushion negative influences (e.g., support and autonomy, career prospects) (see also [19, 21]). The underlying reasoning expects high work demands to imply increased exhaustion, while a lack of work resources leads to an increase in disengagement among workers [33].

In the first group, we distinguish five categories of performed *work tasks* according to Spitz-Oener [34]: non-routine manual, routine manual, routine cognitive, non-routine interactive and non-routine analytic (see Table 1). The individual task composition of these five categories reflects the work activities. It therefore points to different potential health complaints; e.g., a high share of manual tasks may imply physical complaints, while being requested to perform non-routine interactive tasks may be psychologically stressful. In the second group, the *job requirements*, we separate performance from demand: Performance comprises prescribed work implementation and the minimum performance requirements. In addition, we consider whether there are increased performance requirements, such as making improvements or being confronted with new tasks. We further regard requirements that demand parallel management of different processes with a high degree of distraction (multitasking), working towards strict deadlines and performance pressure, and how often individuals have to push their performance limits at work. We assume that a high content of challenging job requirements has a negative impact on both physical and mental health.

**Table 1:** A comprehensive characterisation of health-related job aspects

Job requirements	Work tasks	Working conditions
**Performance specifications** - Prescribed work implementation - Prescribed minimum performance - work fast **High performance requirements** - familiarize with new tasks - improve existing procedures - things you have not learned or you do not master **Repeating operations** - same operations are repeated in every detail **Coordination effort** - strong deadline or performance pressure - disturbed or interrupted at work - keep an eye on different processes simultaneously **Performance limit** - push themselves to the performance limit	**Non-routine manual** - repairing, refurbishing - entertaining, accommodating, preparing food - nursing, caring, healing - protecting, guarding, patrolling, directing traffic **Routine manual** - manufacturing, producing goods and commodities - monitoring, control of machines, plans, technical processes - transporting, storing, shipping - cleaning, removing waste, recycling **Routine cognitive** - measuring, testing, quality control - purchasing, producing, selling - gathering information, investigating, documenting **Non-routine interactive** - advertising, marketing, public relations - training, instructing, teaching, educating - providing advice and information **Non-routine analytic** - organizing, planning and preparing work processes (not own) - developing, researching, constructing	**Physical activities** - Working standing up - Lifting heavy loads - Working in a stooped or kneeling position **Stressful environmental conditions** - Smoke and dust, cold, heat, wetness, dirt, bright light or darkness, noise **Shift work** (Dummy) **Workplace atmosphere** - Part of the working community - Help and support from colleagues/direct superiors **Poor information flow** - You do not receive all the information you need to carry out your job properly - Not being informed in time about far-reaching decisions, changes or plans for the future **Self determination** - Plan and schedule work yourself - Influence on the assigned workload - Decide when to take a break

*Notes:* Allocation of tasks according to [34]. See Table A.2 in the appendix for a detailed definition and description of variables

*Source:* [35, 36]. Own allocation

The last group comprises the *working conditions*. These include information about physical exertion and aspects of environmental influences under which work is carried out. Physically stressful work and the work environment may both physically but also mentally affect a person. The working atmosphere constitutes an important part of the working conditions. We capture it by focusing on teamwork in the workplace, mutual support and permanent exchange of information. The degree of self-determination in the workplace is measured by the possibility of determining the workload individually (see Table 1).

### Data

For the empirical analysis, we use the BIBB/BAuA Labour Force Survey 2012 and 2018 [35, 36] provided by the Federal Institute for Vocational Education and Training (BIBB) and the Federal Institute for Occupational Safety and Health (BAuA). The survey gathers data on working conditions and requirements as well as the acquisition and exploitation of occupational knowledge in the German labour market every 6 to 7 years since 1979. The core labour force is defined as employed persons from the age of 15 years without apprentices and without marginally employed persons (paid work for at least 10 h a week). However, we have to restrict our analysis to the recent two waves of 2012 and 2018 since only these waves provide consistent information on both work characteristics and health status.^1^ Each wave contains approximately 20,000 individuals, and the data sets are merged into a single database for empirical analysis.

We will examine whether there are differences in work tasks, job requirements and working conditions between migrants and native Germans within an occupation. The data provide information on citizenship and mother tongue only. We therefore define individuals’ migration background according to Oldenburg et al. [11]: (1) *Foreigners* are individuals without German citizenship, whereas (2) *Germans with a migration background* have a second foreign citizenship in addition to German citizenship, or they are in possession of German citizenship but learned a foreign mother tongue during childhood. (3) *Native Germans* (or Germans without a migration background) are persons with German citizenship, and no further foreign mother tongue was learned during childhood or no second citizenship is in place. The distinction between migrants and native Germans approximates the official definition of persons with a migration background by the Federal Statistical Office of Germany [13].^2^ As classified, we combine *Foreigners* and *Germans with a migration background* into *persons with a migration background* (hereafter: migrants) for further analysis. We are aware that the group of *persons with a migration background* is extremely heterogeneous, imposing a limitation for deriving specific policy implications. Unfortunately, the data do not allow a differentiated analysis of migrants due to the low number of observations per group, and we are not aware of any more suitable data available at the moment. Hence, the coarse distinction we use in the analysis provides a useful starting point to identify potential differences. Of course, it should be augmented by future research taking the heterogeneity of the groups considered explicitly into account.

In the following analyses, only persons of labour force age (15 to 64 years) who are not employed by the military are considered. We conduct wage trimming at both ends of the hourly wage distribution by 1% each to exclude highly incomprehensible combinations of wage and actual working time. Based on this, our estimation sample contains 38,187 observations, of which 35,364 are *native Germans* (92.6%) and 2,823 are *migrants* (7.4%). Relative to the official numbers, migrants are underrepresented in our data.^3^ Based on data of the German micro-census, survey weights are provided for the BIBB/BAuA data according to the characteristics of gender, age, education, and German/non-German, among others [35]. These survey weights minimize selection bias and correct for deviations from the previous year’s micro-census, thus obtaining a representative sample of the economically active population aged 15 and over in Germany used in our empirical analysis.

### Descriptive statistics

**Fig. 2:**
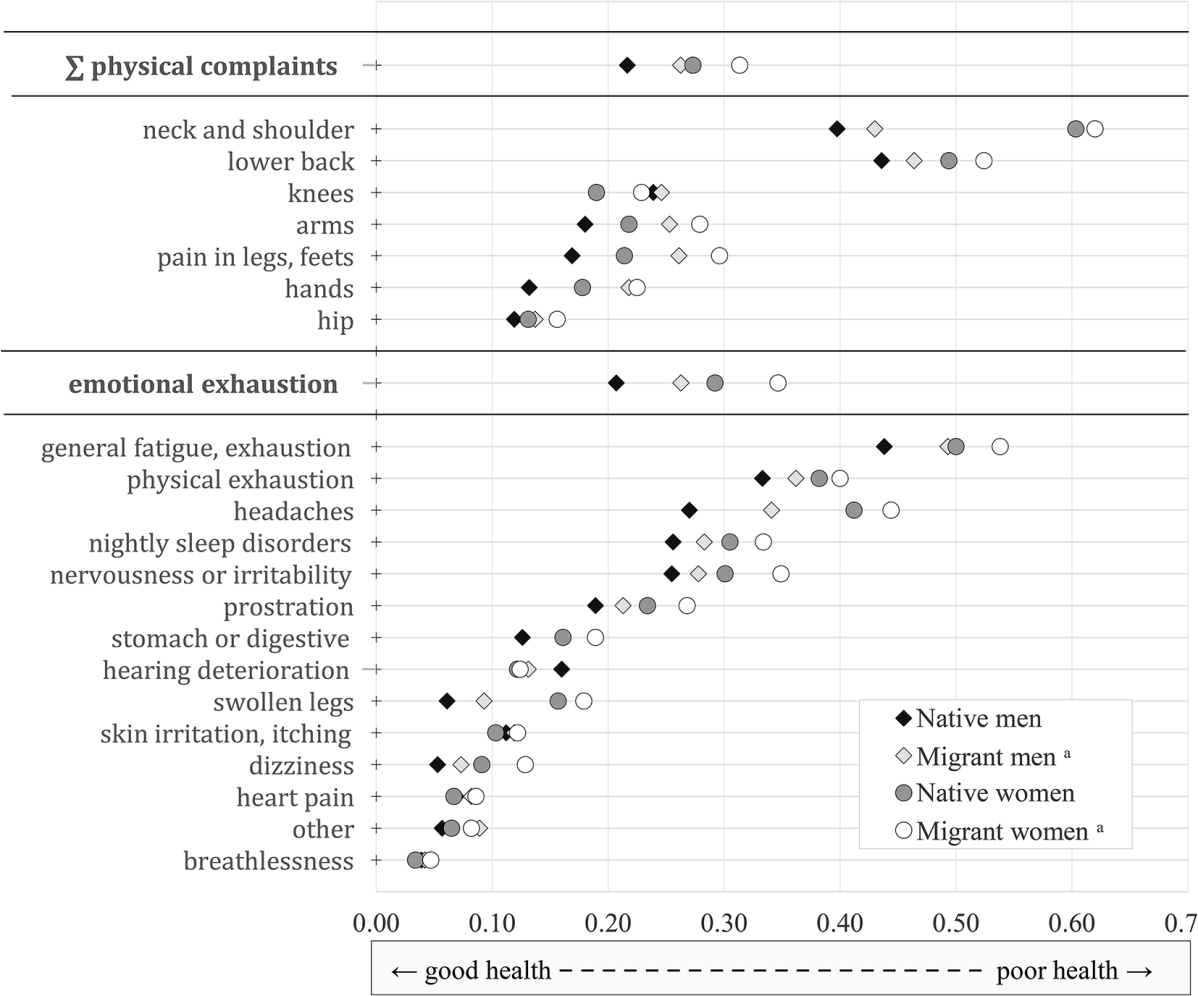
Means of physical and mental complaints by gender and migration background (2012, 2018). *Notes*: Survey weights are considered to counteract sample bias. Sorted by weighted mean. Persons in labour force age only. Average number of observations: 17,020 male native Germans, 1,420 male migrants, 18,290 female native Germans, 1,390 migrant women. Physical complaints are an aggregate of afflictions of the lower back, neck and shoulder, hip, arms, hands, knees, legs or feet (musculoskeletal disorders). Emotional exhaustion is used as a proxy for mental health. a) Foreigners and Germans with migration background. *Source* [35, 36]. Own calculations

The general health condition is reported on a 5-point Likert scale, which we reversed from poor (0), not so well (0.25), well (0.5), very good (0.75), to excellent (1) for better interpretation (see Table A.2 in the appendix for detailed variable definitions).^4^ Women report an overall worse state of health than men (see Table 2). The overall health status is rated slightly higher by migrant men (0.584) than by native men (0.573), while it does not differ significantly between native women (0.553) and migrant women (0.545). For a more comprehensive and detailed description, Fig. 2 considers information on whether physical and mental health complaints have occurred during work or on working days in the last 12 months. A first notable finding is that although the frequency of symptoms is comparable, women consistently report symptoms more often than men; and migrants consistently report symptoms more frequently than native Germans do. Only knee complaints and hearing deterioration occur more frequently among men than among women. The most common symptoms mentioned are complaints in the neck and shoulder, as well as in the lower back, general fatigue and headaches. Strong relative differences between migrants and native Germans can be found in naming physical complaints during work (as an aggregate of afflictions of the lower back, neck and shoulder, hip, arms, hands, knees, legs or feet) which occur significantly more frequently among migrants, both for women (+ 15%) and for men (+ 21%).^5^ The relative differences between migrants and native Germans are even greater for mental health problems in the form of emotional exhaustion, both for women (+ 19%) and for men (+ 27%). The disproportionate mentioning of complaints by migrants compared to native Germans is also apparent in almost all other categories (see Table A.1 in the appendix).

Occupations are primarily characterised by performed work tasks and required qualifications. The survey participants were asked about a number of different *tasks* and how often they perform these activities at work: frequently, sometimes or never. A task is included in the participants’ job description if it is performed “frequently”. The standardized values (i.e. with mean 0 and standard deviation normalized to 1) for work tasks differ between migrants and native Germans: Independent of gender, migrants carry out significantly more routine and in tendency also more non-routine manual tasks as well as non-routine analytic tasks than native Germans do, on average. In contrast, native Germans perform significantly more non-routine interactive tasks, while there were no significant differences in routine cognitive tasks (see Table 2). These differences can indicate a relatively low degree of substitutability in interactive tasks for migrants and native Germans, due to different labour market-relevant skills. Potential reasons may be language differences [38] and/or (the admission of) qualifications obtained abroad [39].

**Table 2:** Work-related descriptive statistics by gender and migration background (2012, 2018)

Z-standardized variables	Migrant ^a^ men	Native men	Diff. Mig.-Nat. men	Migrant ^a^ women	Native women	Diff. Mig.-Nat. women
*Health*						
General health status	0.12	0.07	0.05*	-0.07	-0.03	-0.04
Physical complaints	0.10	-0.09	0.19***	0.31	0.14	0.17***
Emotional exhaustion (0|1)	-0.03	-0.15	-0.12***	0.16	0.04	0.12***
*Work tasks *						
Non-routine manual	0.15	0.00	0.15***	0.14	0.05	0.09***
Routine manual	0.38	0.31	0.07**	0.01	-0.01	0.02
Routine cognitive	-0.01	0.00	-0.01	-0.08	-0.06	-0.02
Non-routine interactive	-0.28	-0.22	-0.06**	-0.12	0.03	-0.15***
Non-routine analytic	0.13	-0.03	0.16***	-0.02	-0.11	0.09***
*Job requirements*						
Quantity performance	0.25	0.05	0.20***	0.12	0.02	0.10***
High performance requirements	-0.04	0.02	-0.06**	-0.26	-0.14	-0.12﻿***
Coordination efforts	-0.20	-0.07	-0.13***	-0.39	-0.07	-0.32﻿***
Working at performance limit	-0.01	-0.02	0.01	-0.25	-0.05	-0.20﻿***
Repeating operations	0.08	-0.02	0.10***	0.20	0.14	0.06**
*Working conditions*						
Physical activities	0.29	0.26	0.03	0.07	0.03	0.04
Stressful environmental conditions	0.43	0.48	0.05	-0.16	-0.17	0.01
Shift work (0|1)	0.29	0.13	0.16***	0.07	0.00	0.07﻿***
Working climate	-0.24	-0.01	-0.23***	-0.08	0.01	-0.09﻿***
Insuf. information transfer	0.05	0.05	0.00	-0.16	-0.08	-0.08﻿***
Self determination	-0.13	-0.03	-0.10***	-0.19	-0.15	-0.04*
Obs.	1,427	17,046		1,396	18,318	

*Notes* * *p* < 0.1, ** *p* < 0.05, *** *p* < 0.01 − Survey weights are considered to counteract sample bias. Persons in the labour force age only. We treat ordinal-scaled variables as continuous

a) Foreigners and Germans with a migration background

*Source* * [35, 36]. Own calculations

We further use standardized values for *job requirements* and *working conditions* originally surveyed by 4-point Likert scales, with respondents reporting whether certain conditions occur “frequently” (4), “sometimes” (3), “rarely” (2) or “never” (1). Regarding *job requirements*, migrants are significantly more often confronted with quantity specifications, monotonous assignments and/or repetitive operations. In contrast, migrants are less often entrusted with tasks that have demanding high performance requirements or a high degree of coordination and responsibility. These are even rarer for migrant women (see Table 2). The *working conditions* of migrants and native Germans do not differ significantly in terms of physically stressful environmental influences and in terms of physical activities. This is not surprising given the high level of work safety regulations in Germany. However, migrants perform their work significantly more often in shift work. There are also clear differences in the way migrants and native Germans evaluate the workplace atmosphere. Migrants rate the working climate significantly worse in the sense that they feel less involved in the working community, and migrants are also less likely to state that they are allowed to determine their own workflow (see Table 2).^6^ Our descriptive analysis points to significant differences in tasks, (perceived) requirements and (perceived) conditions at work between migrants and native Germans. By how far these differences interact and for quantifying their (relative) importance, we present the results of a multivariate analysis in the next section.

### Empirical strategy

To empirically analyse how much the state of health can be attributed to the individual labour market situation, we specify an empirical model following our suggested conceptual framework. We regress work-related health on a set of variables characterising work tasks, work requirements, working conditions and the socio-economic situation of the individual. Our theoretical considerations above implied gender-specific perceptions of health and work tasks. We thus consider estimation of separate models by gender and migration background for each of our self-reported health indicators: general health (SRH$\:)$, physical health (MSD), and mental health (EMX). The econometric notation used for all three indicators and model variations is the following (exemplary for SRH):

1 $$\begin{aligned} SRH & = \alpha + SOCIO{\beta _1} + WORK{\beta _2} + TASK{\beta _3} \\&+ REQ{\beta _4} + COND{\beta _5} + FSY{\beta _6} + \mu \\ \end{aligned}$$

where $\:\varvec{S}\varvec{R}\varvec{H}$ is a vector of the standardized self-reported general health status. $\:\varvec{S}\varvec{O}\varvec{C}\varvec{I}\varvec{O}$ is a matrix of socio-demographic characteristics (i.e., age, age squared, gender, vocational education level, foreign citizenship, foreign mother tongue, marital status and the presence of children in the household).^7^ The variables contained in $\:\varvec{W}\varvec{O}\varvec{R}\varvec{K}$ capture the individual scope of work and occupational status: real working hours (continuous), real working hours squared, job position (4 size categories, from high to low), hourly wage (continuous), firm size dummies (5 size categories, from large to small), and occupational dummies using the German Classification of Occupations 2010 (KldB) at the 2digit level (see Table A.2 in the appendix for detailed variable definitions). Based on our characterisation of job contents, we consider the three groups of factors: The matrix $\:\varvec{T}\varvec{A}\varvec{S}\varvec{K}$ contains five standardized task categories. $\:\varvec{R}\varvec{E}\varvec{Q}$ represents a set of job requirements regarding different work performance specifications, while $\:\varvec{C}\varvec{O}\varvec{N}\varvec{D}$ includes a set of the working conditions to which the employees are exposed (see Sect. 3). The matrices $\:\varvec{R}\varvec{E}\varvec{Q}$ and $\:\varvec{C}\varvec{O}\varvec{N}\varvec{D}\:$comprise a set of standardized survey questions further compiled to standardized summary indicators. The compositions of the indicators for job requirements and working conditions are given in Table A.2 in the appendix. To take differences in the share of migrants across regions and over time into consideration, we control for federal states and survey years ($\:\varvec{F}\varvec{S}\varvec{Y}$). $\:{\beta\:}_{1}$ to $\:{\beta\:}_{6}$ denote the corresponding coefficient vectors, $\:\mu\:$ is the i.i.d. vector of the error terms. We specify the estimation models on work-related physical and mental complaints analogously. Physical complaints represent a set of musculoskeletal disorders (MSD). Mental health describes emotional exhaustion (EMX), see Fig. 2 for an overview of the contained afflictions.

## Results

### General health status

Table 3 shows the main estimation results with regard to individuals’ general health status (SRH). When considering socio-economic characteristics only (specifications 1 to 4), the relationships between individual characteristics and the self-reported general health status are comparable independent of gender and migration background. In line with theory, age has the expected significant negative correlation with health, whereas a higher level of vocational education shows a strong positive correlation with health status. A partnership is also positively related to general health.

However, additional consideration of work attributes and controls for regional and time trends (specifications 5 to 8) mitigates the relationship between individual characteristics and health status. The statistical significance of the difference between the respective coefficients is indicated by the p-values from Chi-squared tests. The test can be used to examine if coefficients in two multivariate regression estimations differ. Based on the fact that we compare migrants and native Germans within the same occupation and socio-economic status, the influence of work-related factors on general health should not differ or should at least be nearly the same. Obviously, the results indicate that this it is not the case. Individuals’ age remains significant only for native Germans, which indicates that socio-demographic and work characteristics may have a different and stronger impact on migrants’ than on native Germans’ health. The level of vocational education and the job position are more conducive to health for native Germans and less for migrants. Moreover, workers’ occupational status indicates a substantial explanation for general health status only for female native Germans, whereas no significant influence can be identified for the other groups. Contrasting this evidence with the theoretical reasoning discussed above has an important implication: Our empirical findings confirm the implications of the theory only for native Germans, but they do not so for migrants. For migrants, the coefficients even have opposite signs of those for natives.

**Table 3:** Regression results on general health conditions

Ordinary least squares (OLS)	Men		Women		Men			Women		
**Depended variable**: Self-reported health (z-values)	Migrant ^a^	Native	Migrant ^a^	Native	Migrant ^a^	Native	Prob > chi2	Migrant ^a^	Native	Prob > chi2
	(1)	(2)	(3)	(4)	(5)	(6)	(5)/(6)	(7)	(8)	(7)/(8)
*Individual characteristics*										
Age	-0.047*	-0.057***	-0.047*	-0.043***	-0.026	-0.050***	0.391	-0.026	-0.034***	0.781
Age^,^ squared	0.000	0.000***	0.000	0.000***	0.000	0.000***	0.416	0.000	0.000**	0.932
Education: Vocational training	0.037	0.031	-0.032	0.207***	-0.035	-0.002	0.779	-0.137	0.127**	0.049
Education: Advanced training	0.034	0.152***	0.134	0.345***	-0.160	0.010	0.346	-0.059	0.179**	0.227
Education: University degree	0.306***	0.386***	0.330***	0.470***	-0.081	0.060	0.344	-0.117	0.208**	0.034
Partnership-Dummy	0.111	0.070***	0.097	0.107***	0.036	0.008	0.767	-0.017	0.068***	0.353
Children in the household	-0.053	0.010	-0.285***	0.015	-0.062	-0.004	0.540	-0.270***	0.002	0.005
*Work characteristics*										
Real working hours					-0.023	0.005	0.139	-0.048***	-0.005	0.004
Real working hours, squared					0.000	-0.000	0.330	0.001***	0.000	0.009
Job pos.: skilled worker					0.015	0.045	0.768	0.026	0.027	0.994
Job pos.: highly qualified empl.					0.022	0.099**	0.593	0.016	0.112***	0.369
Job pos.: specialist					0.270	0.085	0.393	0.622	0.115	0.092
Hourly wage					0.002	0.009***	0.225	0.011*	0.007***	0.505
Firm size, 5 categories					X	X		X	X	
KldB, 2-digit level					X	X		X	X	
*Work tasks (z-values)*										
Non-routine manual					-0.011	0.014	0.587	0.033	0.015	0.726
Routine manual					-0.018	0.004	0.619	-0.128**	-0.002	0.014
Routine cognitive					0.057	-0.000	0.184	0.022	-0.016	0.349
Non-routine interactive					-0.067	0.004	0.135	-0.052	0.027**	0.061
Non-routine analytic					-0.025	-0.008	0.692	0.056	0.008	0.265
*Job requirements (z-values)*										
High performance requirements					0.076	0.014	0.197	0.022	0.010	0.809
Repeating operations					-0.018	-0.033***	0.744	-0.050	-0.016	0.416
Coordination efforts					-0.027	-0.044***	0.701	-0.094**	-0.019	0.123
Quantity performance					0.017	-0.008	0.537	-0.029	-0.019	0.843
Working at performance limit					-0.072*	-0.108***	0.380	-0.102**	-0.148***	0.361
*Working conditions (z-values)*										
Physical activities					0.011	-0.019	0.589	-0.075	-0.071***	0.995
Stressful environmental conditions					-0.134**	-0.077***	0.282	0.005	-0.115***	0.063
Shift work (0|1)					-0.059*	0.012	0.021	-0.012	0.003	0.687
Working climate					0.114***	0.122***	0.836	0.078**	0.120***	0.237
Insuf. information transfer					-0.078**	-0.093***	0.684	-0.023	-0.076***	0.215
Self determination					0.125***	0.081***	0.315	0.038	0.053***	0.726
*Control*										
Federal states					X	X		X	X	
Survey years					X	X		X	X	
Constant	1.309***	1.518***	1.066**	0.890***	0.826	1.260***		0.202	1.025***	
Obs.	1,416	16,953	1,387	18,227	1,122	13,915		1,078	15,249	
adj. R^2^	0.051	0.077	0.072	0.059	0.202	0.175		0.190	0.185	

*Notes:* * *p* < 0.1, ** *p* < 0.05, *** *p* < 0.01 − Survey weights are considered to counteract sample bias. Persons in labour force age only. For a detailed description of variable definitions see Table A.2 in the appendix

a) Foreigners and Germans with migration background

*Source*: [35, 36]. Own calculations

Since we include standardized work-related factors in our models, we can directly compare the influence of *work tasks*, *work requirements* and *working conditions* on the general state of health. The estimated effects reveal notable differences in the magnitude of influence of the three central groups of work-related factors between migrants and native Germans. With regard to *job requirements*, we observe that working frequently at the performance limit has the strongest significant negative impact on individuals’ health status of all regarded work-related factors: An increase of one standard deviation (SD) decreases the general health status by 0.07 (migrant men) to 0.15 SD (native women), on average. The strength of this impact is highly plausible, as it can negatively influence all other job requirements. Native Germans show an even stronger burden of working at the performance limit when job requirements are considered separately (see Table A.4 in the appendix). Moreover, performing repeated operations is significantly stressful only for native Germans (-0.03 SD), high performance requirements tend to show a positive (but not significant) relationship with health, whereas coordination efforts (0.09 SD for migrant women) and quantity performance negatively correlate with individuals’ general health condition (see Table 3). *Work tasks* have at best a small impact on health. Although not significant, the influence relating to the work tasks performed is consistently higher for migrants, indicating a higher relevance for this group (see Table 3). The more negative influence of non-routine interactive tasks on migrants’ health is emphasized by the statistically significant differences (p-values of Chi-squared tests). Beyond that, if work tasks are regarded separately, the results confirm that non-routine interactive tasks are in tendency more burdensome for migrants which may be due to language-based interactions (see Table A.4 in the appendix).

According to our estimation results, *working conditions* are the group of work-related factors with the overall strongest influence on health. While we generally find little difference between the groups, working conditions appear to have a greater impact on women’s health. Working in a stressful environment significantly leads to poorer health of 0.08 to 0.13 SD, except for migrant women. The results also confirm the negative association between a high level of physical burden and health status but to a lesser extent than expected. In the same way, information asymmetries approximated by insufficient information transfer within the firm are detrimental to employee health (-0.04 SD for migrant women, -0.09 SD for native men). In contrast, positive interpersonal interactions in the workplace increase the likelihood of good health. Hence, a good workplace atmosphere has the strongest positive and group-independent impact on the health of all work-related factors: an increase of one SD improves the general health status by between 0.08 SD (migrant women) and 0.12 SD (native Germans). This is supported by a significant positive impact of self-determination at work (from 0.04 SD for native women to 0.13 SD for migrant men) (see Table 3). Overall, educational level and job position seem to be more beneficial for health for native Germans than for migrants. There are significant differences between migrants and native Germans, predominantly among women.

### Physical complaints

To allow a better understanding of whether factors affect certain subdomains of health differently, we present the results of separate models using the same model specifications as the general health status. A constituent part of general health in the labour market is *physical health*. In our case, physical complaints comprise musculoskeletal disorders − here, as an aggregate of afflictions of the lower back, neck and shoulder, hip, arms, hands, knees, legs or feet. Physical complaints during work are comparatively more frequently reported by migrants than by native Germans, and women disclose a higher exposure than men (see Fig. 2 above).

No less surprisingly, physical health problems are strongly promoted by physical activities (about 0.05 SD) as well as physically stressful environmental conditions at work (0.03 to 0.07 SD). However, migrant men are more physically burdened by stressful environmental conditions than native Germans, whereas among women it is the exact opposite (see Table A.5 in the appendix). Furthermore, the frequent performance of routine manual and non-routine analytic tasks more strongly promotes musculoskeletal disorders among migrants than among native Germans. While job requirements such as repeated operations place a significant health burden on native Germans, quantity performances affect the physical health of migrant men more negatively than those of native men. On the other hand, a good working atmosphere overall not only enhances general health conditions (indirectly) but also diminishes physical health problems. However, indicators of working conditions do not significantly influence physical health of migrant women at all. In addition, migrants’ individual skills and work characteristics have less impact on their physical health than to those of native Germans do. It is noticeable that a higher job position of native Germans comes – at least in tendency – with a lower occurrence of physical health problems. Besides, a higher professional degree of males is associated with fewer physical complaints only among native Germans, but not among migrants (see Table A.5 in the appendix). This circumstance indicates that the level of education of migrants is reflected less in their physical health than in that of natives.

### Emotional exhaustion

Emotional exhaustion occurs comparatively more often among migrants than among native Germans, and women report a higher exposure than men (see Fig. 2 above). With regard to the triggering factors, Table A.6 in the appendix reports the estimation results of our preferred linear probability model specification for the years 2012 and 2018. The most important factor promoting emotional exhaustion in all groups is frequent working at the performance limit: An increase of one SD raises the occurrence of emotional exhaustion by 5.4 to 8.6 ppts. At this, the psychological burden of quantity performances and performing non-routine manual tasks (for women) as well as coordination efforts and performing non-routine interactive tasks (for men) is clearly higher for migrants than for native Germans. Non-routine tasks generally seem to be more conducive to emotional exhaustion. On the other hand, a good working atmosphere significantly reduces emotional exhaustion. This positive impact is more pronounced for native Germans than for migrants (-5.7 to -6.7 ppts) and thus has an equally large but opposite impact as working at the performance limit. For migrants, the impact is only about − 3 ppts. Furthermore, adverse working conditions, such as a physically stressful work environment, have a significant negative influence on mental health for all individuals (about 3 ppts), but especially for migrant women (8 ppts). In contrast, self-determined work shows no influence on emotional exhaustion of women, but there is a particularly strong impact for migrant men (-5 ppts). Regarding a higher job position, the major difference in terms of impact on mental health is that it makes emotional exhaustion less likely for native Germans, but not so for migrants. Indeed, we observe almost no impact for migrants. Overall, working conditions and job requirements seem to have less influence on the mental health of migrant women (see Table A.6 in the appendix).

## Discussion

The results of our model add new and detailed empirical evidence to the established literature [8, 18, 21, 23] by revealing different health impacts among migrants and native Germans with further heterogeneity by gender.^8^ The circumstance that age has no significant impact on migrants’ health when work characteristics are taken into account indicates an unequal health burden due to work. The plausibility of this interpretation is further strengthened by a weaker influence of the occupational education level and the job position on migrants’ health compared to native Germans. The empirical results contradict the theoretical explanations by Burgard and Lin [8] of a decreasing health burden with higher education and occupational status for the case of migrants. They give a strong indication for a potentially unequal treatment of workers with respect to ethnic background in Germany.

Differences in workload between migrants and native Germans lie predominantly in the tasks and working conditions, but less in the job requirements themselves. The health burden of the tasks performed at work is considerably more severe on the health of migrants than on native Germans. However, of all the work-related factors, the influence of working frequently at the performance limit is the most negative health burden. Its importance should not be underestimated, as it can severely affect mental and physical health in the long run if there is not enough (time for) recovery. A balanced management of workload and recovery contributes to reducing health stress among workers (see “effort-recovery model” by Meijman and Mulder [40]). On the other hand, with regard to the “job demands-resources model” [33], the strong positive influence of working conditions should also be given equal consideration in order to preserve workers’ health. This implication is clearly supported by our evidence on work atmosphere: The results indicate a significant contribution to maintaining health during times of increased workloads. The fact, that working conditions in general have a much weaker impact on the health of migrant women could be due to their less sensitive perception of these influences. Nevertheless, further research is required on this issue. By and large, however, there are elements in the control of the employer to counteract the workload and health burden of its employees. In terms of physical health, our findings confirm that physical activities and physically stressful environmental conditions at work promote musculoskeletal disorders. In contrast to physical health, mental health is predominantly facilitated by working at the performance limit. Differences in mental health between migrants and native Germans are mainly reflected in the perception of the working climate. Our empirical results further confirm a larger susceptibility to emotional exhaustion of women; reasons are discussed in several studies, e.g. by Posig and Kickul [41].

Our empirical analysis focuses solely on the direction of the effect of workload on associated health. It is therefore necessary to further refine and extend the framework to the dynamic context of health formation. Future considerations may provide further explanations with regard to the heterogeneity of the groups. Despite the large set of characteristics incorporated in our empirical analysis, a more detailed characterisation may contain aspects like level of physical activity, smoking, healthy nutrition, and use of preventive health services [42]. Low socio-economic status is generally associated with adverse expression of these activities. Such detailed characterisation may allow to identify and to quantify factors of key importance, which may be addressed explicitly in health prevention measures. Moreover, additional aspects that affect health may be supplemented to the model, e.g., work engagement and work attitudes (behaviour) or health-influencing activities in the private sphere. Nevertheless, our detailed empirical model of socio-economic and workplace characteristics is able to depict the different work-related health consumption of individuals.

We contrast comparable groups within particular working circumstances, where the consumption of health should be approximately the same. However, differences in the effect size of work-related factors on health between migrants and native Germans within the same delimitation indicate a different consumption of health. A possible reason for this could be the unequal treatment of migrants and native Germans in the workplace. In addition, our empirical results further indicate a stronger perception of workload and related health afflictions. Hence, behavioural differences and differences in perception may also lead to the revealed inequality in health status. Although this would mitigate disadvantage or even discrimination against migrants as reasons for inequality, the unequal states still imply differences in productivity, well-being and the efficient use of individual capabilities in the labour market. Gaps in general health status between migrants and native Germans may additionally be driven by differences in their health investments. A number of studies recognises a lower healthcare utilisation among migrants [5, 43].

Implications of our findings are diverse. Differences in health status could be countered by customized company health management and adequate preventive health measures by the employer. On the health consumption side, efforts should continue to reduce the burden of working at performance limits and to improve the conditions of the working environment. Progress in these dimensions is expected to reduce socio-economic inequalities in health [7]. Furthermore, the observed disparities in health investments should be an incentive for better communication and/or promotion of healthcare utilisation by migrants. Migrants should be enabled to make greater use of health services for reducing the risk of social decline. Policies to improve social and health status, and access to healthcare among migrants and ethnic minorities are essential to reduce ethnic inequalities in health [27]. For this purpose, barriers to accessing healthcare must be identified in a first step, and be removed in a second step. In this regard, economic incentives may be given larger weight. They could easily be designed in such a way that decisions are made in favour of health improvement (i.e. nudges); to become effective, they should take ethnic (and/or socio-economic) differences in perception – and in utilisation – into account.

## Conclusions

This study examined the unequal workload and its impact on the health status of migrants and natives in Germany based on an integrated framework for consideration of work tasks, job requirements and working conditions. We incorporated a detailed characterisation of work-related factors and their influence on the self-reported health of employees in the empirical analysis.

Our analyses show that migrants systematically report a worse state of health than natives. Our results further reveal some differences in the workload of migrants and natives, but no evidence of a different treatment of migrants in the workplace. Hence, even within comparable groups within particular working circumstances, where the consumption of health should be approximately the same, the negative health effects of work-related conditions are stronger on people with a migration background than they are on natives. This holds both for physical complaints and emotional exhaustion.

Importantly, we found that women systematically reported worse health conditions than men. With regard to these gender-specific differences, our results show that working conditions appear to have a greater impact on women’s health. Women also disclose a higher exposure of physical complaints during work than men.

Our findings clearly reveal the fundamental value of promoting human capital to address and reduce economic and health disparities. For this reason, they support the general need to improve investments in human capital as a precondition to strengthen income security, social protection, and living conditions and for reducing income and health inequalities [44]. Measures for a selective promotion of migrants to counteract possible disadvantages (affirmative actions) should be considered with great caution, as they may involve a selective granting of an advantage in turn.

Our study attempts to raise attention on this topic, but more research is required for recommendation of specific interventions. Additional research is furthermore needed for understanding what the explanatory mechanism at work is exactly. Potentially, promising avenues for future research may be a more detailed look into the heightened precariousness and exposure to labour exploitation of migrants [45], into their heightened exposure to specific hazards within the same occupation compared to the native population [46], and into the diversity and heterogeneity of the group of persons with a migration background.

### Electronic supplementary material

Below is the link to the electronic supplementary material.

Supplementary Material 1

## Data Availability

The data that support the findings of this study are available at the “Bundesinstitut für Berufsbildung” (BIBB). Data sources can be found in the reference list under the corresponding institution.

## Footnotes

1 The data provide a subjective assessment of work requirements, working conditions and individual health status. See Table A.2 in the appendix for exact variable definitions.

2 A distinction between direct (1st generation) and indirect (2nd generation) migration background is not possible due to the lack of information on individual migration history.

3 In the years 2012 and 2018, the official share of employed *persons with a migration background* in Germany was 17.4% respectively 21.9% [13, 37]. In 2018, the unemployment rate of *persons with a migration background* (5.8%) was twice as high as that of native Germans (2.6%) [13], so the share of this group in the BIBB/BAuA Labour Force Survey must inevitably be lower.

4 We treat ordinal-scaled variables as continuous.

5 The aggregation of eight afflictions results in a discrete distribution in nine values.

6 Further descriptive statistics on individual and work-related characteristics can be found in Appendix Table A.3.

7 Due to estimation of separate models by gender and migration background (defined by “foreign nationality” and “foreign mother tongue”, see above) these variables are not included in the following analyses.

8 A limitation of our study is the quite coarse definition of persons with migration background. The data at hand do not provide information to grasp the heterogeneity of this group. Particularly, they do not allow to distinguish persons with migration background in necessary detail to unveil potentially heterogeneous effects, both within and between different groups of origin, migration experience, legal status, further socio-economic characteristics etc. However, if better data will become available a detailed analysis recognising the heterogeneity of persons with migration background is warranted.
