# Supplementary material for: Workload disparities and their role in the health of migrants and natives in Germany
Source: BMC Public Health. 2024 Aug 9;24:2164. doi: 10.1186/s12889-024-19606-3 (PMC11316351; doi:10.1186/s12889-024-19606-3)
Supplement: Supplementary file 1 — Supplementary Material 1 [file 12889_2024_19606_MOESM1_ESM.docx]

Workload Disparities and Their Role in the Health of Migrants and Natives in Germany

Kai Ingwersen

Leibniz University Hannover, Institute of Economic Policy, Königsworther Platz 1, D-30167 Hannover, Germany,

e‑mail: ingwersen@wipol.uni-hannover.de, ORCID: 0000‑0001‑7219‑384X

Stephan L. Thomsen

Leibniz University Hannover, Institute of Economic Policy, Königsworther Platz 1, D-30167 Hannover, Germany,

e-mail: thomsen@wipol.uni-hannover.de, phone: +49 511 762 14596 (✉ **corresponding author**),
ORCID: 0000‑0002‑9677‑9199

This version: June 22, 2024

# **Appendix**

**Table A.1**: Means of health complaints by gender and migration background (2012, 2018)

|  |  | **Men** |  |  |  | **Women** |  |  |
| --- | --- | --- | --- | --- | --- | --- | --- | --- |
|  | Native | Migrant ^a^ | Diff. |  | Native | Migrant ^a^ | Diff. |  |
| **general health status** | 0.57 | 0.58 | 0.01 | * | 0.55 | 0.55 | -0.01 |  |
| **∑ physical complaints** | 0.22 | 0.26 | 0.05 | *** | 0.27 | 0.31 | 0.04 | *** |
| neck and shoulder | 0.40 | 0.43 | 0.03 | ** | 0.60 | 0.62 | 0.02 |  |
| lower back | 0.44 | 0.46 | 0.05 | ** | 0.49 | 0.52 | 0.03 | ** |
| knees | 0.24 | 0.25 | 0.01 |  | 0.19 | 0.23 | 0.04 | *** |
| arms | 0.18 | 0.25 | 0.07 | *** | 0.22 | 0.28 | 0.06 | *** |
| pain in legs, feets | 0.17 | 0.26 | 0.09 | *** | 0.21 | 0.30 | 0.08 | *** |
| hands | 0.13 | 0.22 | 0.09 | *** | 0.18 | 0.23 | 0.05 | *** |
| hip | 0.12 | 0.14 | 0.02 | ** | 0.13 | 0.16 | 0.03 | *** |
| general fatigue, exhaustion | 0.44 | 0.49 | 0.06 | *** | 0.50 | 0.54 | 0.04 | *** |
| physical exhaustion | 0.33 | 0.36 | 0.03 | ** | 0.38 | 0.40 | 0.02 |  |
| headaches | 0.27 | 0.34 | 0.07 | *** | 0.41 | 0.44 | 0.03 | ** |
| nightly sleep disorders | 0.26 | 0.28 | 0.03 | ** | 0.31 | 0.33 | 0.03 | ** |
| nervousness or irritability | 0.26 | 0.28 | 0.02 | * | 0.30 | 0.35 | 0.05 | *** |
| **emotional exhaustion** | 0.21 | 0.26 | 0.06 | *** | 0.29 | 0.35 | 0.05 | *** |
| prostration | 0.19 | 0.21 | 0.02 | ** | 0.23 | 0.27 | 0.03 | *** |
| stomach or digestive | 0.13 | 0.16 | 0.03 | *** | 0.16 | 0.19 | 0.03 | *** |
| hearing deterioration | 0.16 | 0.13 | -0.03 | *** | 0.12 | 0.12 | 0.00 |  |
| swollen legs | 0.06 | 0.09 | 0.03 | *** | 0.16 | 0.18 | 0.02 | *** |
| skin irritation, itching | 0.11 | 0.12 | 0.01 |  | 0.10 | 0.12 | 0.02 | ** |
| dizziness | 0.05 | 0.07 | 0.02 | *** | 0.09 | 0.13 | 0.04 | *** |
| heart pain | 0.07 | 0.08 | 0.01 | * | 0.07 | 0.09 | 0.02 | *** |
| other | 0.06 | 0.09 | 0.03 | *** | 0.07 | 0.08 | 0.02 | ** |
| breathlessness | 0.04 | 0.04 | 0.00 |  | 0.03 | 0.05 | 0.01 | ** |
| Average No. of Obs. | 17,022 | 1,423 |  |  | 18,290 | 1,391 |  |  |

*Notes:* Survey weights are considered to counteract sample bias. Sorted by weighted mean. Persons in labour force age only. We treat ordinal-scaled variables as continuous. The general health status is expressed by a self-reported health scale (0-1). Physical complaints are an aggregate of afflictions of the lower back, neck and shoulder, hip, arms, hands, knees, legs or feet (musculoskeletal disorders). Emotional exhaustion is used as a proxy for mental health.
a) Foreigners and Germans with migration background.

*Source*: [35, 36]. Own calculations.

**Table A.2**: Variable definitions

| **Variable** | **Definition** |
| --- | --- |
| **Dependent variables** |  |
| General health status | self‑reported health: poor (1), not so well (2), good (3), very good (4), excellent (5) |
| Physical complaints | Mean of the frequent occurrence (yes=1, no=0) of the following “musculoskeletal disorders” in the last 12 months during work or on workdays: (1) lower back, (2) neck and shoulder, (3) arms, (4) hands, (5) hip, (6) knee, (7) swollen legs, (8) pain in legs or feets |
| Mental health | Frequent occurrence (yes=1, no=0) of “emotional exhaustion” in the last 12 months during work or on workdays. |
| **Independent variables** |  |
| *Individual characteristics* |  |
| Age | Age at time of survey (starting from 15 years of age) |
| Labour force age | Yes=1 (age from 15 to 64 years)  No=0 (age below 15 or 65 years and older) |
| Partnership | Yes=1 (married, civil partnership)  No=0 (single, divorced, widow) |
| Children | Yes=1 (children in the household)  No=0 (no children in the household) |
| *Origin* |  |
| Foreigners | Individuals without a German citizenship |
| Germans with migration background | German citizens with a second foreign citizenship; or  German citizens but learned a language other than German as a mother tongue during childhood. |
| Native Germans | Individuals with German citizenship and no further foreign mother tongue than German was learned during childhood and no second citizenship is in place. |
| *Education* |  |
| Educational level  (Dummy) | 1. Without professional qualification 2. Vocational training 3. Advanced training 4. University degree |
| *Occupational status* |  |
| Job Position  (Dummy) | 1. Labourer, freelancer, lower level civil servants 2. Skilled worker, journeyman, middle level civil servants 3. Highly qualified employee, upper-level civil servants 4. Foreman, self-employed, senior-level civil servants |
| Real working hours | Average actual weekly working hours, including regular overtime, additional work, standby duty |
| Hourly wage | $\frac{Gross monthly earnings from work}{Actual weekly working hours * 4.35}=gross hourly wage$ $Weeks per month=\frac{365.25/12}{7}=4.35$ |
|  |  |
| Firm size  (Dummy) | 1. Micro: 0-9 employees 2. Small: 10-49 employees 3. Medium: 50-249 employees 4. Large: 250-999 employees 5. Huge: 1000+ employees |
| KldB | German classification of occupations (Klassifikation der Berufe 1992, KldB), 2-digits |

**Continuation of Table A.2**

| *Job requirements* |  |
| --- | --- |
| Quantity specification | In your work, how often does it happen that …  … you are required to carry out your work in every detail? ^a^  … you are prescribed a certain minimum performance or time to do a certain job? ^a^  … you have to work very quickly? ^a^ |
| Performance requirement | In your work, how often does it happen that …  … you are confronted with new tasks, which you first have to think about and get used to? ^a^  … you improve existing procedures or try something new? ^a^  … things are demanded of you which you have not learned or which you have not mastered? ^a^ |
| Repeating operations | In your work, how often does it happen that …  … that one and the same operation is repeated in every detail? ^a^ |
| Coordination effort | In your work, how often does it happen that …  … you have to work under strong deadline or performance pressure? ^a^  … you are disturbed or interrupted at work? ^a^  … you have to keep an eye on different types of work or processes at the same time? ^a^ |
| Performance limit | In your work, how often does it happen that …  … you have to go to the limits of your ability to perform? ^a^ |
| *Work tasks* |  |
| According to Spitz-Oener [34] | Only if the activity is performed "frequently", then the mean value of the assigned tasks is calculated taking into account the number of assigned tasks (Range: 0‑1). ^b^  non-routine manual   - repairing, refurbishing \| entertaining, accommodating, preparing food \| nursing, caring, healing \| protecting, guarding, patrolling, directing traffic   routine manual   - manufacturing, producing goods and commodities \| monitoring, control of machines, plans, technical processes \| transporting, storing, shipping \| cleaning, removing waste, recycling   routine cognitive   - measuring, testing, quality control \| purchasing, producing, selling \| gathering information, investigating, documenting   non-routine interactive   - advertising, marketing, public relations \| training, instructing, teaching, educating \| providing advice and information   non-routine analytic   - organizing, planning and preparing work processes (not own) \| developing, researching, constructing |
| *Working conditions* |  |
| Physically stressful working conditions | - Working while standing ^a^ - Lifting and carrying loads ^a^ - Working in a stooped, squatting, kneeling position or working overhead ^a^ |
| Shift work | - Yes=1 (working in shifts) - No=0 (no shift work) |
| Physically stressful environmental conditions | - Work in smoke, dust or under gases, vapours ^a^ - Work in cold, heat, wet, damp or draughty conditions ^a^ - Working with oil, grease, dirt, grime ^a^ - Work in bright light or in poor or weak lighting ^a^ - Working under noise ^a^ |
| Working atmosphere | How often …  … does it happen that you feel part of a community at your workplace? ^a^  … do you find the cooperation between you and your work colleagues to be good? ^a^  … do you get help and support for your work from colleagues when you need it? ^a^  … do you get help and support for your work from your direct supervisor when you need it? ^a^ |
| Poor information flow | How often does it happen that …  … you are not informed in time about drastic decisions, changes or plans for the future? ^a^  … you do not receive all the information you need to carry out your work properly? ^a^ |
| Self determination | How often does it happen that …  … you can plan and schedule your own work yourself? ^a^  … you have influence on the amount of work assigned to you? ^a^  … you can decide for yourself when to take a break? ^a^ |

1. frequently (1), sometimes (0.25), rarely (0.1), never (0).
2. frequently (1), sometimes (0), never (0) − Use only if the value is "frequently".

*Source*: [35, 36]

**Table A.3**: Descriptive statistics on individual and work-related characteristics (2012, 2018)

|  | Native men | Migrant ^a^ men | Diff. M‑N men |  | Native women | Migrant ^a^  women | Diff. M‑N women |  |
| --- | --- | --- | --- | --- | --- | --- | --- | --- |
| *Individual characteristics* |  |  |  |  |  |  |  |  |
| Age | 43.67 | 40.37 | -3.30 | *** | 43.63 | 39.65 | -3.98 | *** |
| Partnership | 0.56 | 0.62 | 0.06 | *** | 0.55 | 0.57 | 0.02 |  |
| Children in the household | 0.59 | 0.62 | 0.03 | ** | 0.68 | 0.66 | 0.02 |  |
| *Education* |  |  |  |  |  |  |  |  |
| Education: No occupational training | 0.06 | 0.19 | 0.13 | *** | 0.08 | 0.19 | 0.11 | *** |
| Education: Vocational training | 0.59 | 0.47 | -0.12 | *** | 0.62 | 0.46 | -0.16 | *** |
| Education: Advanced training | 0.11 | 0.05 | -0.06 | *** | 0.05 | 0.02 | -0.03 | *** |
| Education: University degree | 0.24 | 0.28 | 0.04 | *** | 0.25 | 0.32 | 0.07 | *** |
| *Work-related characteristics* |  |  |  |  |  |  |  |  |
| Real working hours | 43.16 | 41.75 | -1.41 | *** | 33.92 | 32.15 | -1.77 | *** |
| Job position | 2.29 | 2.13 | -0.16 | *** | 2.02 | 1.88 | -0.16 | *** |
| Job tenure | 13.54 | 10.02 | -3.52 | *** | 12.03 | 7.75 | -4.28 | *** |
| Hourly wage | 18.00 | 18.11 | 0.11 |  | 15.29 | 14.32 | -0.97 | *** |
| *Firm size* |  |  |  |  |  |  |  |  |
| Firm size: micro | 0.18 | 0.18 | 0.00 |  | 0.21 | 0.24 | 0.03 | ** |
| Firm size: small | 0.22 | 0.22 | -0.01 |  | 0.29 | 0.31 | 0.02 |  |
| Firm size: medium | 0.25 | 0.25 | -0.01 |  | 0.24 | 0.21 | -0.03 | *** |
| Firm size: large | 0.17 | 0.14 | -0.03 | *** | 0.14 | 0.13 | -0.01 |  |
| Firm size: huge | 0.18 | 0.22 | 0.04 | *** | 0.11 | 0.11 | 0.00 |  |
| Obs. | 17,046 | 1,427 |  |  | 18,318 | 1,396 |  |  |

*Notes*: * p<0.1, ** p<0.05, *** p<0.01 − Survey weights are considered to counteract sample bias. Persons in labour force age only. We treat the ordinal-scaled variables as continuous.

a) Foreigners and Germans with migration background.

*Source*: [35, 36]. Own calculations.

**Table A.4**: Regression results on general health status separated by work tasks, job requirements and working conditions (2012, 2018)

| **Depended variable:** | Men | | Women | | Men | | Women | | Men | | Women | |
| --- | --- | --- | --- | --- | --- | --- | --- | --- | --- | --- | --- | --- |
| **Self-reported health (z-values)** | Migrant ^a^ | Native | Migrant ^a^ | Native | Migrant ^a^ | Native | Migrant ^a^ | Native | Migrant ^a^ | Native | Migrant ^a^ | Native |
|  | (1) | (2) | (3) | (4) | (5) | (6) | (7) | (8) | (9) | (10) | (11) | (12) |
| *Individual characteristics* |  |  |  |  |  |  |  |  |  |  |  |  |
| Age | -0.029 | -0.064*** | -0.044 | -0.047*** | -0.025 | -0.060*** | -0.036 | -0.041*** | -0.035 | -0.051*** | -0.031 | -0.038*** |
| Age^,^ squared | 0.000 | 0.000*** | 0.000 | 0.000*** | 0.000 | 0.000*** | 0.000 | 0.000*** | 0.000 | 0.000*** | 0.000 | 0.000** |
| Education: Vocational training | 0.018 | -0.008 | -0.111 | 0.172*** | 0.058 | -0.026 | -0.086 | 0.176*** | -0.042 | 0.019 | -0.086 | 0.125** |
| Education: Advanced training | 0.023 | 0.018 | -0.028 | 0.232*** | -0.007 | 0.009 | 0.001 | 0.251*** | -0.113 | 0.015 | -0.054 | 0.183*** |
| Education: University degree | 0.155 | 0.112** | 0.028 | 0.277*** | 0.169 | 0.080 | 0.088 | 0.285*** | -0.093 | 0.077 | -0.006 | 0.214*** |
| Partnership-Dummy | 0.100 | 0.033 | 0.062 | 0.083*** | 0.090 | 0.028 | 0.065 | 0.081*** | 0.028 | 0.008 | 0.040 | 0.063** |
| Children in the household | -0.131 | 0.006 | -0.216** | 0.010 | -0.104 | 0.015 | -0.244*** | 0.029 | -0.037 | -0.013 | -0.258** | -0.007 |
| *Work characteristics* |  |  |  |  |  |  |  |  |  |  |  |  |
| Real working hours | 0.006 | 0.002 | -0.035*** | -0.012*** | 0.007 | 0.008** | -0.031** | -0.004 | -0.028 | 0.003 | -0.049*** | -0.009** |
| Real working hours, squared | -0.000 | 0.000 | 0.000*** | 0.000*** | -0.000 | -0.000 | 0.000** | 0.000** | 0.000 | -0.000 | 0.001*** | 0.000* |
| Job pos.: skilled worker | -0.029 | 0.048 | -0.011 | 0.051* | -0.040 | 0.073** | 0.008 | 0.082*** | 0.011 | 0.027 | -0.001 | 0.009 |
| Job pos.: highly qual. employee | 0.024 | 0.144*** | 0.001 | 0.157*** | -0.003 | 0.177*** | 0.015 | 0.206*** | 0.025 | 0.073* | 0.033 | 0.077* |
| Job pos.: specialist | 0.134 | 0.150*** | 0.533*** | 0.198*** | 0.082 | 0.185*** | 0.478*** | 0.187*** | 0.292 | 0.055 | 0.537 | 0.080 |
| Hourly wage | 0.006 | 0.012*** | 0.014** | 0.010*** | 0.007 | 0.013*** | 0.015*** | 0.011*** | 0.002 | 0.008*** | 0.010 | 0.007*** |
| Firm size, 5 categories | x | x | x | x | x | x | x | x | x | x | x | x |
| KldB, 2-digit level | x | x | x | x | x | x | x | x | x | x | x | x |
| *Work tasks (z-values)* |  |  |  |  |  |  |  |  |  |  |  |  |
| Non-routine manual | -0.035 | -0.020 | -0.013 | -0.029** |  |  |  |  |  |  |  |  |
| Routine manual | -0.076* | -0.030** | -0.170*** | -0.058*** |  |  |  |  |  |  |  |  |
| Routine cognitive | 0.070* | 0.002 | -0.010 | -0.024** |  |  |  |  |  |  |  |  |
| Non-routine interactive | -0.024 | 0.005 | -0.049 | 0.015 |  |  |  |  |  |  |  |  |
| Non-routine analytic | -0.003 | -0.009 | 0.062 | 0.009 |  |  |  |  |  |  |  |  |
| *Job requirements (z-values)* |  |  |  |  |  |  |  |  |  |  |  |  |
| Performance requirements |  |  |  |  | 0.120*** | 0.021* | 0.004 | 0.014 |  |  |  |  |
| Repeating operations |  |  |  |  | -0.041 | -0.038*** | -0.074* | -0.022** |  |  |  |  |
| Coordination efforts |  |  |  |  | -0.072 | -0.066*** | -0.078* | -0.047*** |  |  |  |  |
| Quantity performance |  |  |  |  | -0.033 | -0.038*** | -0.063 | -0.053*** |  |  |  |  |
| Working at performance limit |  |  |  |  | -0.101** | -0.142*** | -0.121*** | -0.184*** |  |  |  |  |
| *Working conditions (z-values)* |  |  |  |  |  |  |  |  |  |  |  |  |
| Physical activities |  |  |  |  |  |  |  |  | 0.002 | -0.032* | -0.124** | -0.095*** |
| Environmental conditions |  |  |  |  |  |  |  |  | -0.154*** | -0.093*** | -0.087 | -0.138*** |
| Shift work |  |  |  |  |  |  |  |  | -0.057* | 0.013 | -0.014 | -0.001 |
| Working climate |  |  |  |  |  |  |  |  | 0.121*** | 0.130*** | 0.079** | 0.133*** |
| Insuf. information transfer |  |  |  |  |  |  |  |  | -0.086** | -0.118*** | -0.070* | -0.106*** |
| Self determination |  |  |  |  |  |  |  |  | 0.137*** | 0.085*** | 0.017 | 0.063*** |
| *Control* |  |  |  |  |  |  |  |  |  |  |  |  |
| Federal states | x | x | x | x | x | x | x | x | x | x | x | x |
| Survey years | x | x | x | x | x | x | x | x | x | x | x | x |
|  |  |  |  |  |  |  |  |  |  |  |  |  |
| constant | 0.422 | 1.641*** | 1.111 | 1.162*** | -0.061 | 1.254** | 0.693 | 0.633*** | 1.363* | 1.474*** | 0.418 | 1.272*** |
| Obs. | 1,330 | 16,251 | 1,253 | 17,084 | 1,318 | 16,188 | 1,252 | 17,5017 | 1,140 | 14,087 | 1,108 | 15,477 |
| adj. R^2^ | 0.111 | 0.094 | 0.154 | 0.089 | 0.119 | 0.128 | 0.177 | 0.135 | 0.200 | 0.158 | 0.157 | 0.162 |

*Notes*: * p<0.1, ** p<0.05, *** p<0.01 − Survey weights are considered to counteract sample bias.
a) Foreigners and Germans with migration background.

*Source*: [35, 36]. Own calculations.

**Table A.5**: Regression results on physical complaints (2012, 2018)

| **Ordinary least squares (OLS)** | **Men** | | | **Women** | | |
| --- | --- | --- | --- | --- | --- | --- |
| Depended variable: | Migrant ^a^ | Native | Prob>chi2 | Migrant ^a^ | Native | Prob>chi2 |
| **Musculoskeletal disorders (MSD)** | (1) | (2) | (1)/(2) | (3) | (4) | (3)/(4) |
| *Means (z-values)* | *0.103* | *-0.092* |  | *0.315* | *0.146* |  |
| *Individual characteristics* |  |  |  |  |  |  |
| Age | -0.003 | 0.003** | 0.326 | 0.011 | -0.001 | 0.087 |
| Age^,^ squared | 0.000 | -0.000 | 0.279 | -0.000 | 0.000*** | 0.051 |
| Education: Vocational training | -0.032 | -0.002 | 0.316 | -0.021 | -0.017 | 0.904 |
| Education: Advanced training | 0.026 | -0.023 | 0.242 | 0.010 | -0.041*** | 0.371 |
| Education: University degree | 0.035 | -0.017 | 0.153 | -0.026 | -0.035*** | 0.821 |
| Partnership-Dummy | 0.007 | 0.014** | 0.753 | -0.010 | -0.005 | 0.808 |
| Children in the household | 0.023 | 0.005 | 0.458 | 0.012 | -0.003 | 0.550 |
| *Work characteristics* |  |  |  |  |  |  |
| Real working hours | 0.006 | -0.001 | 0.051 | 0.009** | 0.003*** | 0.104 |
| Real working hours, squared | -0.000 | 0.000 | 0.077 | -0.000* | -0.000*** | 0.200 |
| Job pos.: skilled worker | -0.049** | -0.016** | 0.185 | -0.050* | -0.023*** | 0.307 |
| Job pos.: highly qualified employee | -0.044 | -0.010 | 0.285 | -0.049 | -0.028*** | 0.567 |
| Job pos.: specialist | 0.037 | -0.001 | 0.531 | -0.045 | -0.014 | 0.594 |
| Hourly wage | -0.004** | -0.002*** | 0.170 | -0.001 | -0.002*** | 0.445 |
| Firm size, 5 categories | x | x |  | x | x |  |
| KldB, 2-digit level | x | x |  | x | x |  |
| *Work tasks (z-values)* |  |  |  |  |  |  |
| Non-routine manual | 0.000 | -0.005 | 0.598 | 0.012 | -0.003 | 0.255 |
| Routine manual | 0.019* | 0.002 | 0.105 | 0.039** | 0.009** | 0.039 |
| Routine cognitive | -0.011 | -0.005* | 0.508 | -0.017 | -0.002 | 0.183 |
| Non-routine interactive | 0.008 | 0.003 | 0.686 | 0.010 | -0.001 | 0.303 |
| Non-routine analytic | 0.014 | -0.000 | 0.121 | -0.021* | -0.001 | 0.049 |
| *Job requirements (z-values)* |  |  |  |  |  |  |
| Performance requirements | -0.001 | 0.000 | 0.923 | 0.003 | 0.001 | 0.843 |
| Repeating operations | 0.015 | 0.013*** | 0.876 | 0.004 | 0.010*** | 0.619 |
| Coordination efforts | -0.004 | 0.007** | 0.335 | 0.023** | 0.002 | 0.074 |
| Quantity performance | 0.024* | 0.011*** | 0.247 | 0.020 | 0.013*** | 0.583 |
| Working at performance limit | 0.031*** | 0.025*** | 0.620 | 0.021* | 0.034*** | 0.230 |
| *Working conditions (z-values)* |  |  |  |  |  |  |
| Physical activities | 0.047*** | 0.045*** | 0.910 | 0.043*** | 0.043*** | 0.960 |
| Environmental conditions | 0.069*** | 0.033*** | 0.006 | 0.027* | 0.047*** | 0.211 |
| Shift work | -0.003 | 0.003 | 0.496 | -0.016 | 0.007** | 0.064 |
| Working climate | -0.006 | -0.017*** | 0.200 | -0.009 | -0.018*** | 0.378 |
| Insuf. information transfer | 0.018** | 0.020*** | 0.905 | 0.011 | 0.017*** | 0.562 |
| Self determination | -0.009 | -0.004 | 0.632 | -0.017 | -0.009*** | 0.518 |
| *Control* |  |  |  |  |  |  |
| Federal states | x | x |  | x | x |  |
| Survey years | x | x |  | x | x |  |
|  |  |  |  |  |  |  |
| constant | 0.130 | 0.052 |  | -0.056 | 0.126** |  |
| Obs. | 1,131 | 14,051 |  | 1,093 | 15,304 |  |
| adj. R^2^ | 0.325 | 0.258 |  | 0.273 | 0.287 |  |

*Notes*: * p<0.1, ** p<0.05, *** p<0.01 − Survey weights are considered to counteract sample bias. Persons in labour force age only.
a) Foreigners and Germans with migration background.

*Source*: [35, 36]. Own calculations.

**Table A.6**: Regression results on emotional exhaustion (2012, 2018)

| **Linear Probability Model** | **Men** | | | **Women** | | |
| --- | --- | --- | --- | --- | --- | --- |
| Depended variable: | Migrant ^a^ | Native | Prob>chi2 | Migrant ^a^ | Native | Prob>chi2 |
| **Emotional Exhaustion (EMX)** | (1) | (2) | (1)/(2) | (3) | (4) | (3)/(4) |
| *Means (Dummy)* | *0.263* | *0.207* |  | *0.347* | *0.292* |  |
| *Individual characteristics* |  |  |  |  |  |  |
| Age | 0.014 | 0.012*** | 0.881 | 0.004 | 0.006 | 0.882 |
| Age^,^ squared | 0.000* | -0.000*** | 0.772 | -0.000 | -0.000 | 0.852 |
| Education: Vocational training | -0.039 | 0.028 | 0.157 | 0.028 | -0.022 | 0.404 |
| Education: Advanced training | 0.090 | 0.048** | 0.609 | 0.192 | -0.014 | 0.099 |
| Education: University degree | -0.013 | 0.035 | 0.435 | 0.121* | -0.004 | 0.075 |
| Partnership-Dummy | -0.099** | -0.026** | 0.063 | 0.002 | -0.037*** | 0.351 |
| Children in the household | 0.029 | -0.005 | 0.410 | -0.025 | -0.003 | 0.625 |
| *Work characteristics* |  |  |  |  |  |  |
| Real working hours | -0.003 | -0.002 | 0.880 | -0.003 | 0.003* | 0.369 |
| Real working hours, squared | 0.000 | 0.000 | 0.809 | 0.000 | -0.000 | 0.237 |
| Job pos.: skilled worker | -0.053 | -0.003 | 0.234 | -0.061 | -0.004 | 0.251 |
| Job pos.: highly qualified employee | -0.097* | -0.016 | 0.167 | -0.080 | -0.004 | 0.250 |
| Job pos.: specialist | -0.086 | 0.024 | 0.405 | -0.131 | 0.005 | 0.463 |
| Hourly wage | 0.006* | -0.001 | 0.027 | 0.000 | -0.002** | 0.469 |
| Firm size, 5 categories | x | x |  | x | x |  |
| KldB, 2-digit level | x | x |  | x | x |  |
| *Work tasks (z-values)* |  |  |  |  |  |  |
| Non-routine manual | -0.020 | 0.004 | 0.182 | 0.038 | 0.017*** | 0.399 |
| Routine manual | 0.010 | -0.021*** | 0.073 | -0.004 | -0.003 | 0.962 |
| Routine cognitive | 0.009 | -0.004 | 0.472 | -0.015 | 0.008 | 0.280 |
| Non-routine interactive | 0.037* | 0.017*** | 0.322 | 0.034 | 0.009 | 0.267 |
| Non-routine analytic | 0.015 | 0.008 | 0.721 | 0.009 | 0.004 | 0.814 |
| *Job requirements (z-values)* |  |  |  |  |  |  |
| Performance requirements | 0.004 | 0.009* | 0.770 | 0.005 | 0.006 | 0.997 |
| Repeating operations | 0.035** | 0.006 | 0.091 | -0.013 | -0.020*** | 0.735 |
| Coordination efforts | 0.073*** | 0.031*** | 0.019 | -0.003 | 0.007 | 0.644 |
| Quantity performance | 0.012 | 0.015*** | 0.859 | 0.048** | 0.014*** | 0.152 |
| Working at performance limit | 0.075*** | 0.059*** | 0.379 | 0.055** | 0.086*** | 0.158 |
| *Working conditions (z-values)* |  |  |  |  |  |  |
| Physical activities | -0.008 | -0.015** | 0.772 | -0.016 | -0.010 | 0.836 |
| Environmental conditions | 0.025 | 0.026*** | 0.954 | 0.079*** | 0.033*** | 0.091 |
| Shift work | -0.020 | 0.005 | 0.063 | 0.010 | 0.005 | 0.809 |
| Working climate | -0.022 | -0.057*** | 0.017 | -0.020 | -0.067*** | 0.010 |
| Insuf. information transfer | 0.031* | 0.035*** | 0.782 | 0.034* | 0.047*** | 0.522 |
| Self determination | -0.050*** | -0.018*** | 0.081 | 0.014 | -0.015*** | 0.145 |
| *Control* |  |  |  |  |  |  |
| Federal states | x | x |  | x | x |  |
| Survey years | x | x |  | x | x |  |
|  |  |  |  |  |  |  |
| constant | 0.266 | -0.041 |  | 0.714 | 0.036 |  |
| Obs. | 1,120 | 13,903 |  | 1,078 | 15,239 |  |
| adj. R^2^ | 0.173 | 0.132 |  | 0.128 | 0.162 |  |

*Notes*: * p<0.1, ** p<0.05, *** p<0.01 − Survey weights are considered to counteract sample bias. Persons in labour force age only.
a) Foreigners and Germans with a migration background.

*Source*: [35, 36]. Own calculations.
